# Antimicrobial activity of natural products against MDR bacteria: A scientometric visualization analysis

**DOI:** 10.3389/fphar.2022.1000974

**Published:** 2022-09-26

**Authors:** Yan-Xi Zhou, Xiao-Yu Cao, Cheng Peng

**Affiliations:** ^1^ State Key Laboratory of Characteristic Chinese Medicine Resources in Southwest China, College of Pharmacy, Chengdu University of Traditional Chinese Medicine, Chengdu, China; ^2^ Library, Chengdu University of Traditional Chinese Medicine, Chengdu, China

**Keywords:** antibacterial activity, MDR, natural products, biofilm, scientometric analysis

## Abstract

**Objective:** A growing number of studies have demonstrated the antimicrobial activity of natural products against multidrug-resistant bacteria. This study aimed to apply scientometric method to explore the current status and future trends in this field.

**Methods:** All relevant original articles and reviews for the period 1997–2021 were retrieved from the Web of Science Core Collection database. VOSviewer, a scientometric software, and an online bibliometric analysis platform were used to conduct visualization study.

**Results:** A total of 1,267 papers were included, including 1,005 original articles and 262 reviews. The United States and India made the largest contribution in this field. The University of Dschang from Cameroon produced the most publications. Coutinho HDM, Kuete V, and Gibbons S were key researchers, as they published a great many articles and were co-cited in numerous publications. Frontiers in Microbiology and Antimicrobial Agents and Chemotherapy were the most influential journals with the highest number of publications and co-citations, respectively. “Medicinal plants”, “methicillin-resistant *Staphylococcus aureus*”, “biofilm”, “minimum inhibitory concentration”, and “efflux pumps” were the most frequently used keywords, so these terms are presumed to be the current hot topics. All the included keywords could be roughly divided into four major themes, of which the theme of “natural product development approach” had attracted much attention in recent years. Furthermore, “*Pseudomonas aeruginosa*”, “nanoparticles”, “green synthesis”, “antimicrobial peptides”, “antibiofilm”, “biosynthetic gene clusters”, and “molecular dynamics simulation” had the latest average appearance year, indicating that these topics may become the research hot spots in the coming years.

**Conclusion:** This study performed a scientometric analysis of the antibacterial activity of natural products against multidrug-resistant bacteria from a holistic perspective. It is hoped to provide novel and useful data for scientific research, and help researchers to explore this field more intuitively and effectively.

## Introduction

Infections induced by bacterial pathogens are one of the major causes of morbidity and mortality worldwide (Moore, et al., 2017). Antibiotic treatment is undoubtedly one of the indispensable strategies of modern medicine in order to combat infections or microbes, such as in cancer therapy, organ transplantation, management of preterm babies, or major surgeries (Laxminarayan, et al., 2013). The “golden era” of antibiotic discovery was from the 1930s to the 1960s (Nathan and Cars, 2014). Meanwhile, in the middle of the 20th century, antibiotics were considered as the “wonder drug”. At the time, there was optimism that communicable disorders were nearly conquered (Zaman, et al., 2017). Unfortunately, when microbes are exposed to antibiotics, the likelihood of drug resistance elevates (Rogers Van Katwyk et al., 2020). The underuse, overuse and misuse of antibiotics lead to the development of resistant pathogens (Davies and Davies, 2010). Furthermore, antibiotic resistance is a growing threat to human, animal, and environment health due to the emergence, spread, and persistence of multidrug-resistant (MDR) bacteria or “super bugs” (Aslam, et al., 2018). In general, MDR bacteria are resistant to three or more antibiotics; while *Mycobacterium tuberculosis* strains are extremely resistant to almost all classes of antimicrobials (Subramani, et al., 2017). Ultimately, the discovery of antibiotics has not kept pace with the emergence and development of drug resistant pathogens (Aslam, et al., 2018). A number of important organizations, such as the World Health Organization (WHO), US Centers for Disease Control and Prevention, European Medicines Agency, and World Economic Forum have recognized antibiotic resistance as a global concern (Michael, et al., 2014; Rogers Van Katwyk et al., 2020; Spellberg et al., 2016). It currently threatens tens of millions of lives and poses a major challenge to the global economy and development (Hashem et al., 2022; Rogers Van Katwyk et al., 2020). Consequently, more research and novel sources of antibiotics are urgently needed to overcome resistant problems.

Historically, natural products have played a crucial role in the identification and development of antimicrobial agents (Moloney, 2016). For instance, microbial-derived compounds account for the majority of antibiotic drugs. Decades of purification of antibiotics from microbial sources have identified an estimated 28,000 compounds, about 200 of which have been used directly as drugs. In addition, semi-synthetic improvements to these scaffolds yielded an additional 200–300 drugs, vastly exceeding the number of synthetic antibiotics in clinical use (Wright, 2017). Besides, the phytochemicals of plants are numerous in structure, have few side effects, and most of them have antibacterial activity. As a result, they acquired extra attention in the pharmaceutical industry to enhance the biological activity of existing antibiotics or as a potential source of novel antimicrobial agents active against a variety of pathogens, including MDR bacteria (Jubair, et al., 2021; Salem, et al., 2022). Moreover, β-lactams, tetracycline, macrolides, glycopeptides, lincosamides, sulfonamides, oxazolidinones, and fluoroquinolones are representative classes of antibiotics of the modern era, of which six represent naturally occurring compounds, and only the latter three are obtained entirely by chemical synthesis (Rossiter, et al., 2017). In general, natural-product antibiotics hold a privileged position in this field for the following main reasons: First, they are the products of millions of years of evolution. Through natural selection, they interact with cellular targets with high selectivity and efficiency and avoid drug resistance. Next, unlike numerous synthetic molecules, they are products of evolution that have inherent physicochemical properties necessary to penetrate bacterial cells. Finally, molecular selectivity is a hallmark of a large number of naturally occurring antibiotics, resulting in relatively little widespread toxicity (Wright, 2017).

Scientometrics is the quantitative analysis of bibliographic information using mathematical and statistical approaches. It is usually used to identify overall knowledge frameworks, assess current conditions, and predict future research directions in a certain field (Bornmann and Leydesdorff, 2014; de Castilhos Ghisi et al., 2020). In addition, it can be used to compare the contributions of different countries, institutions, journals, and authors (Ke, et al., 2020). Currently, various visualization tools, such as VOSviewer, CiteSpace, and HistCite have been developed to create knowledge maps and are widely used in multiple scientific research fields, including the medical field (Chen, et al., 2021; Huang, et al., 2021). Given the emergence of MDR bacteria and the importance of natural products in antibiotic drug discovery, numerous studies have been performed in recent years (Hashem and Salem, 2022; Moore, et al., 2017; O’Connell, et al., 2013; Rossiter, et al., 2017). However, as far as we know, no scientometric studies have been reported so far. In view of this, we conducted a scientometric analysis of the antimicrobial activity of natural products against MDR bacteria over a 25-year period from 1997 to 2021. The objective of this study was to identify the major contributors and their collaborative networks, including countries, institutions, and authors, as well as influential journals and references. Additionally, major research clusters, hot topics, and emerging trends in this field are explored.

## Materials and methods

### Data source and search strategy

**FIGURE 1:**
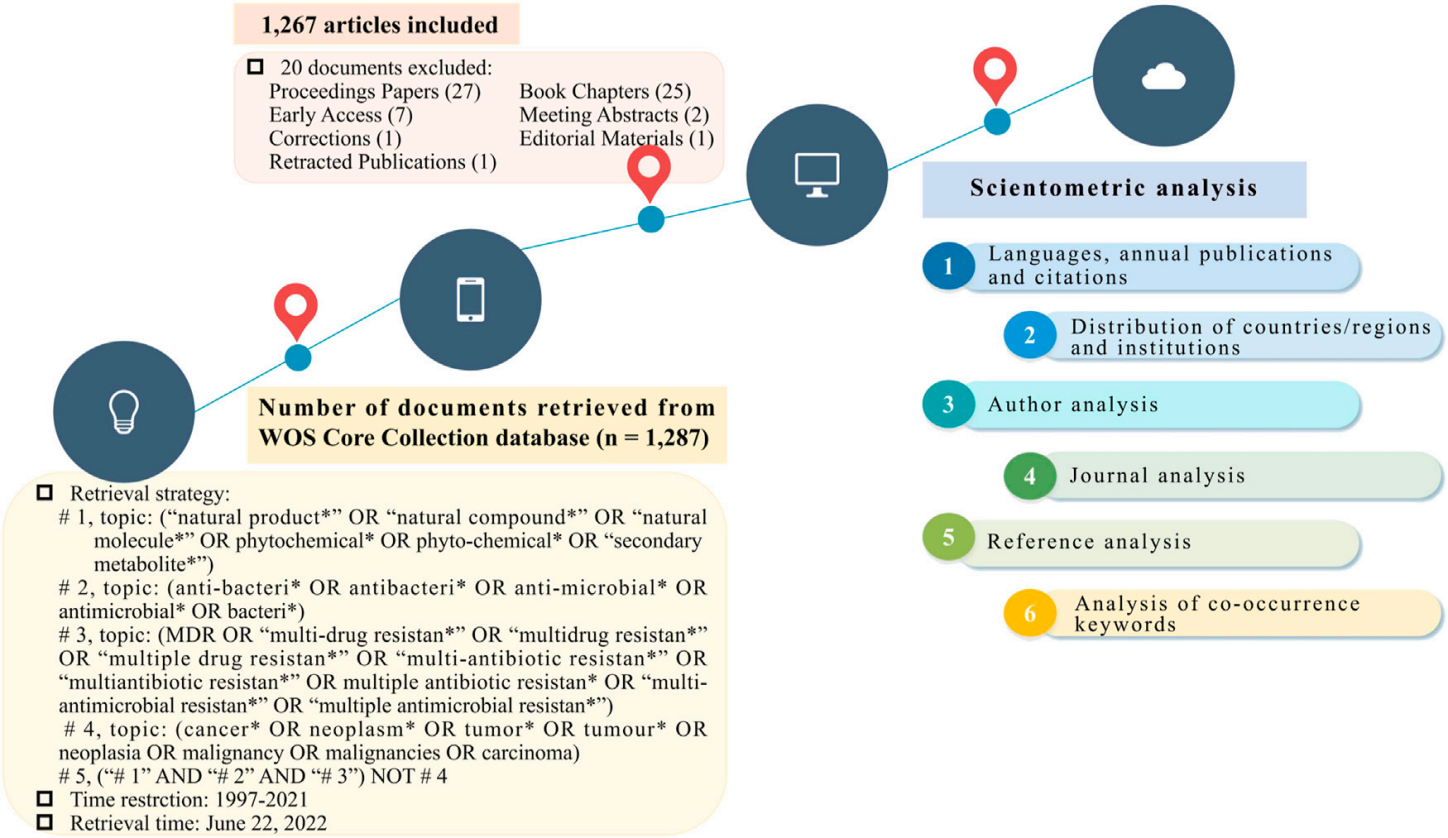
Process of literature identification, screening, and analysis.

Scientometric data were extracted from the Core Collection database of Web of Science (WOS) on 22 June 2022. The retrieval terms and search strategies were used as follows: # 1, topic: (“natural product*” OR “natural compound*” OR “natural molecule*” OR phytochemical* OR phyto-chemical* OR “secondary metabolite*”); # 2, topic: (anti-bacteri* OR antibacteri* OR anti-microbial* OR antimicrobial* OR bacteri*); # 3, topic: (MDR OR “multi-drug resistan*” OR “multidrug resistan*” OR “multiple drug resistan*” OR “multi-antibiotic resistan*” OR “multiantibiotic resistan*” OR “multiple antibiotic resistan*” OR “multi-antimicrobial resistan*” OR “multiple antimicrobial resistan*”); # 4, topic: (cancer* OR neoplasm* OR tumor* OR tumour* OR neoplasia OR malignancy OR malignancies OR carcinoma); # 5, (“# 1” AND “# 2” AND “# 3”) NOT # 4. WOS ignores the hyphen in the search query, for instance, “multiple drug resistan*” would return records that contain the string “multiple-drug resistan*”, “multiple drug-resistan*”, or “multiple-drug-resistan*”. The asterisk (*) is used as a wildcard in the retrieval strategy to represent any group of characters or no character, for example, “multi-drug resistan*” would return “multi-drug resistant” or “multi-drug resistance” (Willem, et al., 2017). Additionally, the time span of the retrieve was from 1997 to 2021, and the publication type was filtered to original articles or reviews. The steps of literature retrieval and selection are presented in Figure 1.

### Data extraction and analysis

According to the search strategy developed above, a total of 1,267 publications were retrieved. By using the export function of WOS, the titles, authors, journals, abstracts and other records of these documents were exported as tab delimited files to VOSviewer software for subsequent analysis. The impact factor (IF) and category partition (Q1, Q2, Q3, and Q4) of each journal were collected from the Journal Citation Reports (JCR) 2021. Data process and cleaning was conducted manually in Microsoft Office Excel 2013. Microsoft Office PowerPoint 2013 was used to create figures on annual research output. Research collaborations between countries are conducted using an online bibliometric analysis platform (https://bibliometric.com/). The rest of the scientometric visualization was performed using VOSviewer 1.6.17. In the network and overlay graph, each node indicates a different parameter, such as countries/regions, journals, authors, and keywords. The different colors of the nodes represent different categories or publication times. The size of the node depends on the number of publications, citations, or occurrences. The link lines between nodes represent the correlation between parameters, the thickness of the lines indicates the strength of the link, and the strength of the link is quantitatively evaluated by total link strength (TLS). In the VOSviewer density graph, the color of the nodes represents the density of the parameter. The darker the color, the more frequently the parameter appears (Huang, et al., 2021; Wu, et al., 2021a; Wu, et al., 2021b).

## Results

### Languages, annual publications and citations

**FIGURE 2:**
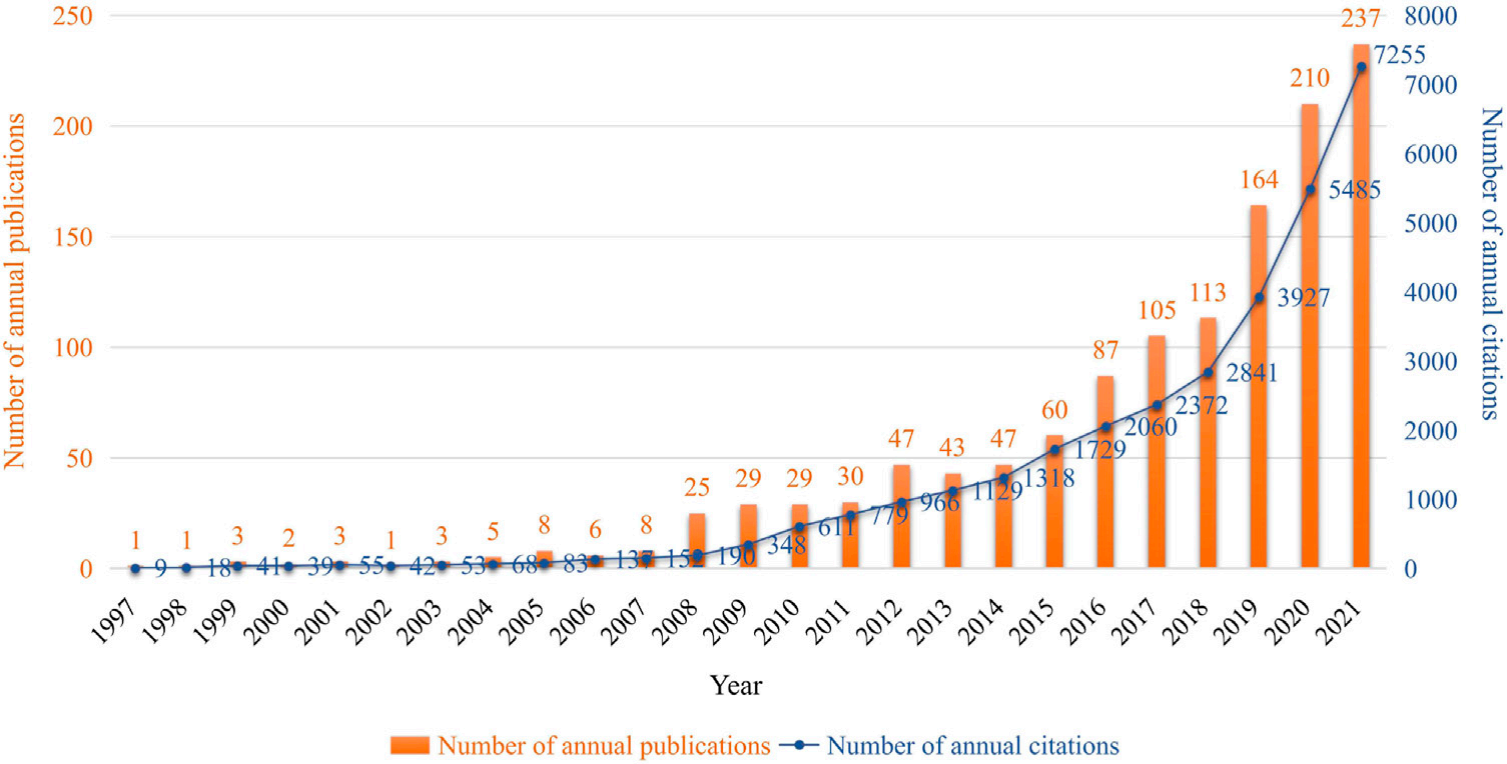
Distribution of annual publications and citations related to resistance of natural products to MDR bacteria from 1997 to 2021.

The literature search resulted in 1,267 related publications including 1,005 original articles and 262 reviews in a period from 1997 to 2021. A total of 5 languages were used in the collected articles, including English (*n* = 1,258, 99.29%), Portuguese (*n* = 6, 0.47%), Chinese (*n* = 1, 0.08%), Japanese (*n* = 1, 0.08%), and Spanish (*n* = 1, 0.08%). The trend of annual publications and citations over the past 25 years is displayed in Figure 2. The number of articles increased from 1 in 1997 to 237 in 2021, and about 65.4% of them were published in the last 5 years. Similar to the annual number of publications, the number of annual citations had an upward trend. The total number of citations for all publications was 34,719 (33,242 times without self-citations), with an average of 27.4 per article, and the H-index of all documents was 87.

### Distribution of countries/regions and institutions

**FIGURE 3:**
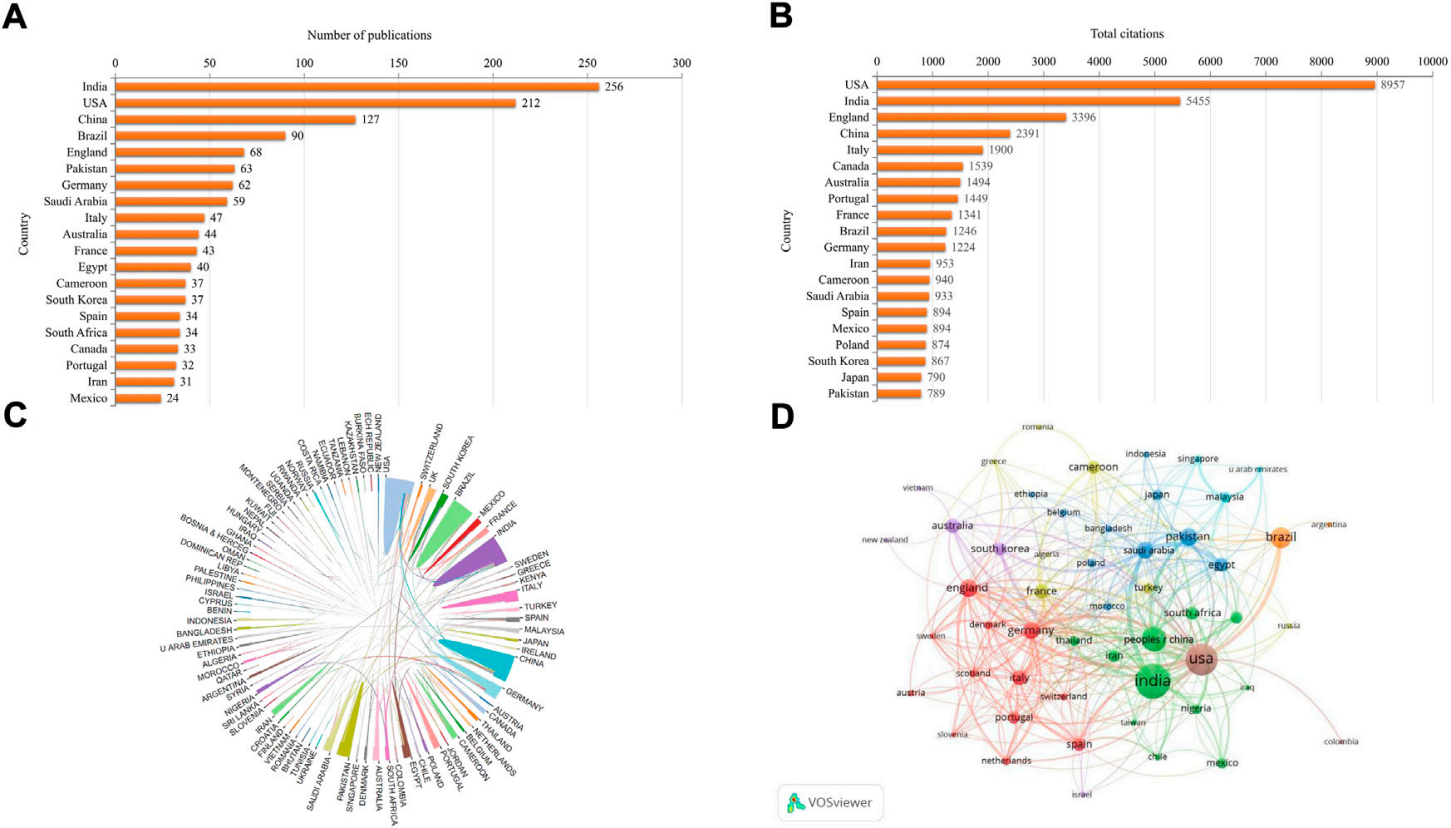
Geographical distribution and international cooperation of countries/regions related to resistance of natural products to MDR bacteria. **(A)** Top 20 countries/regions with the largest number of publications. **(B)** Top 20 countries/regions with the largest number of citations. **(C)** International cooperation among all countries/regions. **(D)** Network visualization map of co-authorship among countries/regions with 5 or more publications.

A total of 95 countries/regions contributed to publications related to resistance of natural products to MDR bacteria. India was the country with the largest output, with 256 papers (20.21%), followed by the United States (212, 16.73%), China (127, 10.02%), Brazil (90, 7.10%), and England (68, 5.37%) (Figure 3A). Articles from the United States had the most citations (8,957 times), followed by India (5,455 times), England (3,396 times), China (2,391 times), and Italy (1,900 times) (Figure 3B). Figure 3C illustrates international cooperation among all countries/regions. The United States demonstrated the most active cooperation, and had close collaborations with India, China, Germany and Canada. Figure 3D displays the country co-authorship network visualization map using VOSviewer. Only countries/regions with 5 or more papers were included, and 52 countries/regions met this threshold. The top five countries with the highest TLS were the United States (TLS = 151), China (TLS = 93), India (TLS = 80), Saudi Arabia (TLS = 77), and Germany (TLS = 74).

**FIGURE 4:**
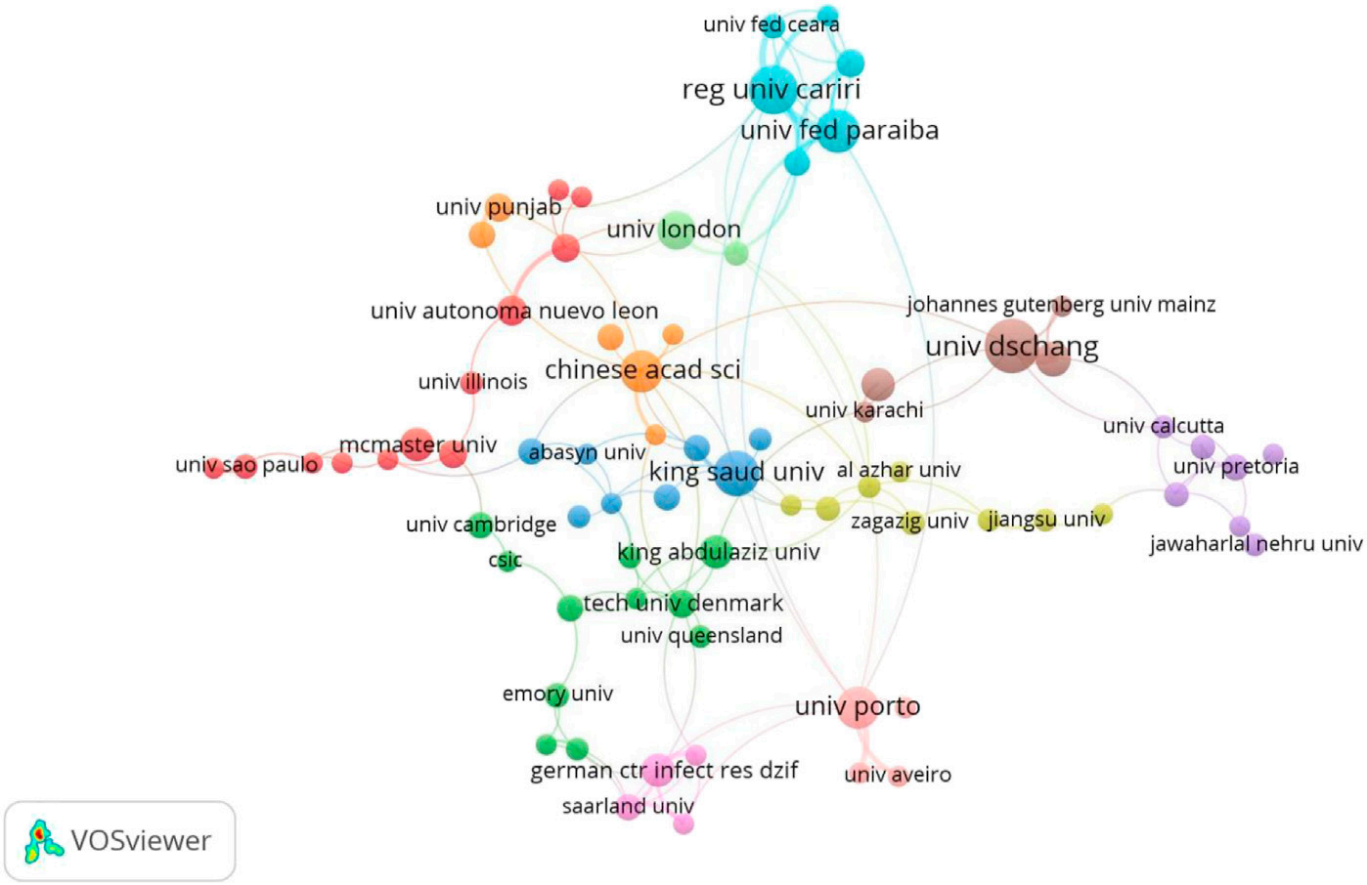
Network visualization map of co-authorship among institutions with 5 or more publications.

In terms of institution analysis, a total of 1,881 institutions contributed to the publications in this filed. The top 10 most prolific institutions are presented in Table 1. The University of Dschang from Cameroon was the leading contributor in terms of number of articles with 33 papers (2.60%), followed by Regional University of Cariri (27, 2.13%), King Saud University (22, 1.74%), Federal University of Paraiba (21, 1.66%), and University of Porto (20, 1.58%). The University of London had the highest citation/publication ratio (83.81) among the top 10 productive institutions. Figure 4 shows the institution co-authorship network visualization map using VOSviewer. Only institutions with 5 or more publications were included, and 83 institutions met this threshold. The five institutions with the highest TLS were Regional University of Cariri (TLS = 33), Federal University of Paraiba (TLS = 28), Universidade Federal do Piaui (TLS = 20), University of Dschang (TLS = 17), and University of Porto (TLS = 16).

**TABLE 1:** The top 10 productive institutions related to resistance of natural products to MDR bacteria.

Rank	Institutions	Country/Region	Documents	% (of 1,267)	Citations	Citations per article	TLS
1	University of Dschang	Cameroon	33	2.60	891	27.00	17
2	Regional University of Cariri	Brazil	27	2.13	368	13.63	33
3	King Saud University	Saudi Arabia	22	1.74	215	9.77	10
4	Federal University of Paraiba	Brazil	21	1.66	234	11.14	28
5	University of Porto	Portugal	20	1.58	1,081	54.05	16
6	Chinese Academy of Sciences	China	19	1.50	363	19.11	14
7	University of London	England	16	1.26	1,341	83.81	4
8	University of Yaounde I	Cameroon	14	1.10	171	12.21	16
9	King Abdulaziz University	Saudi Arabia	13	1.03	205	15.77	5
10	German Center for Infection Research (DZIF)	Germany	12	0.95	112	9.33	13

### Author analysis

**FIGURE 5:**
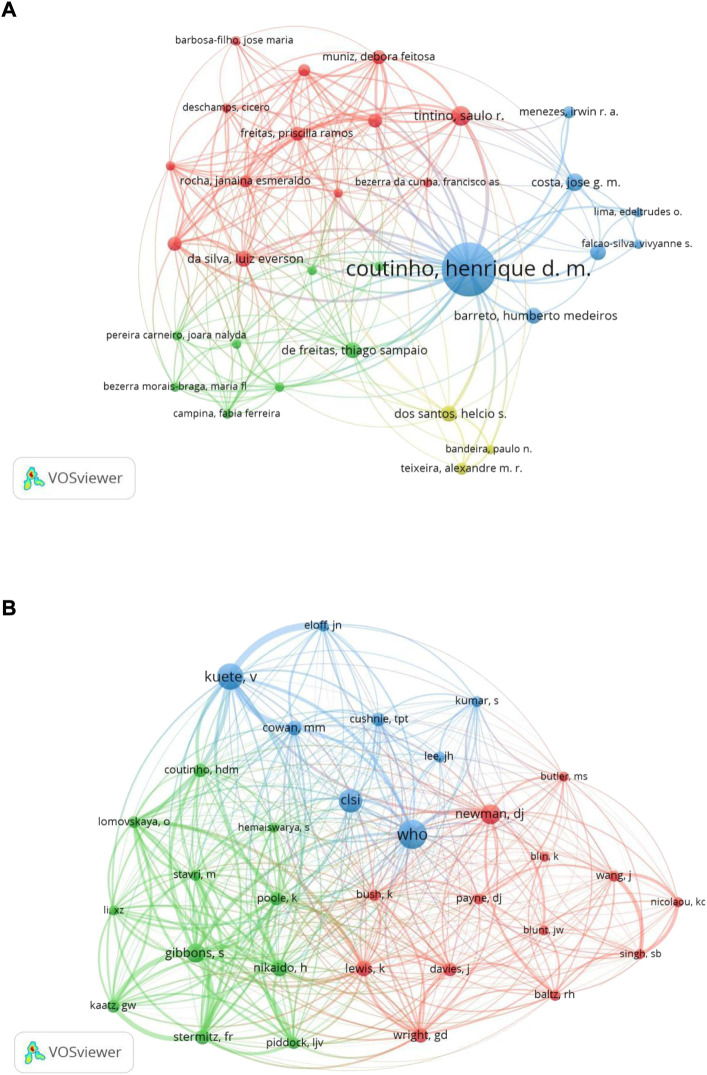
Network visualization map of authors **(A)** and co-cited authors **(B)** related to resistance of natural products to MDR bacteria.

A total of 6,438 authors were involved in the antimicrobial effect of natural products against MDR bacteria. The top 10 authors according to the number of papers contributed 131 papers (10.34%) in this field (Table 2). Coutinho HDM from Brazil published the most articles (33, 2.60%), followed by Kuete V (31, 2.45%) and Gibbons S (12, 0.95%) from Cameroon and England, respectively. In the author co-authorship network visualization map (Figure 5A), only authors with 3 or more papers were included, and 185 authors met the criteria. Coutinho HDM was located at a central position of the co-authorship map and had close cooperation with multiple authors such as Tintino SR, Costa JGM, Dos Santos HS, and De Freitas TS. Furthermore, the co-citation analysis between authors was also performed. Among 45,118 co-cited authors, six authors had co-citations over 100 times. WHO had the most co-citations (230 times) and ranked the first, followed by Kuete V (205 times) and the Clinical and Laboratory Standards Institute (CLSI) (173 times) (Table 2). In the author co-citation network graph (Figure 5B), authors with a minimum number of citations equal to or greater than 50 times were included, and 32 authors reached the threshold. The top five authors with the greatest TLS were Gibbons S (TLS = 791), Kuete V (TLS = 704), Stermitz FR (TLS = 689), WHO (TLS = 618), and Nikaido H (TLS = 573).

**TABLE 2:** The top 10 authors and co-cited authors related to resistance of natural products to MDR bacteria.

Rank	Author	Documents	% (of 1,267)	Citations per article	Country/Region	Co-cited author	Co-citations	TLS
1	Coutinho HDM	33	2.60	8.94	Brazil	WHO	230	618
2	Kuete V	31	2.45	27.94	Cameroon	Kuete V	205	704
3	Gibbons S	12	0.95	43.67	England	CLSI	173	268
4	Mueller R	9	0.71	6.56	Germany	Newman DJ	136	459
5	Wright GD	9	0.71	63.67	Canada	Gibbons S	127	791
6	Pages JM	8	0.63	55.13	France	Nikaido H	101	573
7	Tintino SR	8	0.63	7.88	Brazil	Lewis K	93	512
8	Bolla JM	7	0.55	58.00	France	Stermitz FR	91	689
9	Costa JGM	7	0.55	13.29	Brazil	Wright GD	89	437
10	Fankam AG	7	0.55	44.00	Cameroon	Cowan MM	86	416

### Journal analysis

In total, 505 journals emerged in this field. The 10 journals with the most publications are listed in Table 3. The top 10 active journals published a total of 228 articles, accounting for 18% of all 1,267 articles. *Frontiers in Microbiology* published the most articles (36, 2.84%), followed by *Molecules* (34, 2.68%), *BMC Complementary and Alternative Medicine* (29, 2.29%), *Antibiotics-Basel* (27, 2.13%), and *Marine Drugs* (19, 1.50%). Among the top 10 prolific journals, *Marine Drugs* had the highest IF of 6.085, followed by *Frontiers in Microbiology* (6.064) and *Applied Microbiology and Biotechnology* (5.560). *Applied Microbiology and Biotechnology* had the highest citation/publication ratio (40.07). According to the 2021 edition of the JCR, five, four, and one journals were classified in Q1, Q2 and Q3, respectively.

**TABLE 3:** The top 10 journals and co-cited journals related to resistance of natural products to MDR bacteria.

Rank	Journal	Documents	% (of 1,267)	Citations per article	IF	Quartile in category	Co-cited journal	Co-citations	IF	Quartile in category
1	Frontiers in Microbiology	36	2.84	18.11	6.064	Q1	Antimicrobial Agents and Chemotherapy	2,774	5.938	Q1
2	Molecules	34	2.68	21.26	4.927	Q2	Journal of Natural Products	1,360	4.803	Q1
3	BMC Complementary and Alternative Medicine	29	2.29	27.97	2.838	Q2	Journal of Antimicrobial Chemotherapy	1,093	5.758	Q1
4	Antibiotics-Basel	27	2.13	7.56	5.222	Q1	Journal of Bacteriology	1,090	3.476	Q3
5	Marine Drugs	19	1.50	21.58	6.085	Q1	Journal of Ethnopharmacology	1,090	5.195	Q1
6	Plos One	18	1.42	34.22	3.752	Q2	Proceedings of the National Academy of Sciences of the United States of America	1,090	12.779	Q1
7	Journal of Natural Products	17	1.34	35.00	4.803	Q1	Journal of Antibiotics	1,069	3.424	Q2
8	Scientific Reports	17	1.34	17.00	4.996	Q2	Plos One	906	3.752	Q2
9	Microbial Pathogenesis	16	1.26	10.50	3.848	Q3	Frontiers in Microbiology	887	6.064	Q1
10	Applied Microbiology and Biotechnology	15	1.18	40.07	5.560	Q1	Applied and Environmental Microbiology	794	5.005	Q2

**FIGURE 6:**
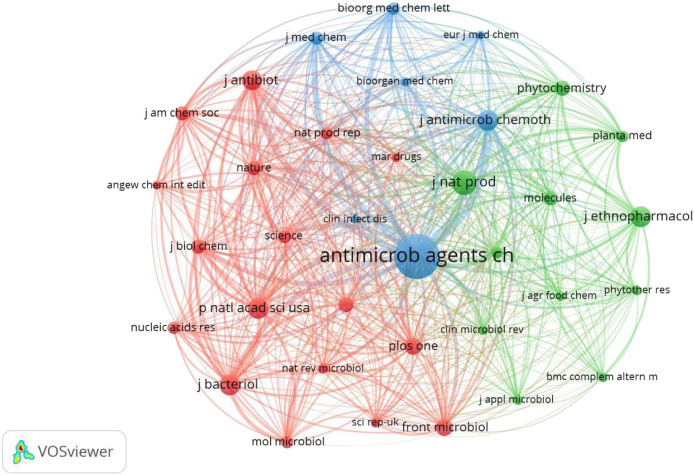
Network visualization map of co-cited journals related to resistance of natural products to MDR bacteria.

In addition, Table 3 presents the top 10 co-cited journals that published related articles. *Antimicrobial Agents and Chemotherapy* had the largest number of co-citations (2,774 times), followed by *Journal of Natural Products* (1,360 times), *Journal of Antimicrobial Chemotherapy* (1,093 times), *Journal of Bacteriology* (1,090 times), *Journal of Ethnopharmacology* (1,090 times), and *Proceedings of the National Academy of Sciences of the United States of America* (1,090 times). The co-citation relationship among journals was visualized in Figure 6. Of the 10,006 co-cited journals, only journals with a minimum number of 400 citations were included, and 35 journals met the threshold. The top five journals with the greatest TLS were *Antimicrobial Agents and Chemotherapy* (TLS = 237,116), *Journal of Natural Products* (TLS = 108,403), *Proceedings of the National Academy of Sciences of the United States of America* (TLS = 102,850), *Journal of Antimicrobial Chemotherapy* (TLS = 95,281), and *Journal of Bacteriology* (TLS = 92,460).

### Reference analysis

**FIGURE 7:**
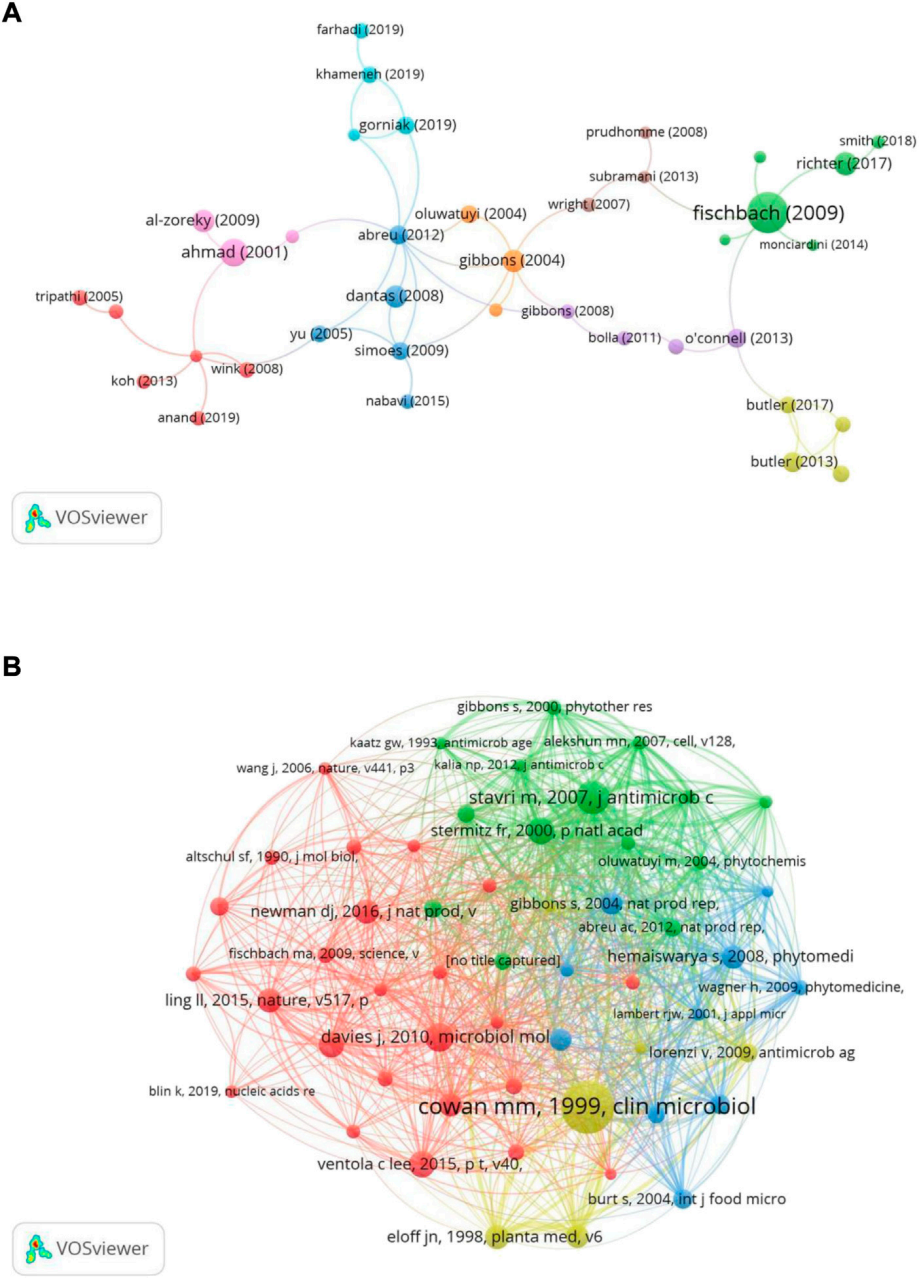
Network visualization map of documents **(A)** and co-cited references **(B)** related to resistance of natural products to MDR bacteria.

According to the document citation analysis, a total of 75 papers were cited more than 100 times (Figure 7A). The basic information of the top 10 most-cited papers is lists in Table 4. These studies were published between 1997 and 2017, six of which were published before 2010. In terms of publication type, 7 were original articles and 3 were reviews. The most cited paper, “Antibiotics for emerging pathogens”, was written by Fischbach and Walsh (2009), with 1,227 citations. The second most cited paper, “Polyphenols as antimicrobial agents”, was written by Daglia (2012), with 779 citations. The third-ranked paper, “Antimicrobial and phytochemical studies on 45 Indian medicinal plants against multi-drug resistant human pathogens”, was written by Ahmad and Beg (2001), with 556 citations. In total, there were 62,965 co-cited references. We presented 54 of these references that were co-cited more than 20 times (Figure 7B). The three references with the largest number of co-citations were written by Cowan (1999) (Clinical microbiology reviews; 86 times), Stavri et al. (2007) (The Journal of antimicrobial chemotherapy; 55 times), and Davies and Davies (2010) (Microbiology and molecular biology reviews; 47 times).

**TABLE 4:** The top 10 most-cited papers related to resistance of natural products to MDR bacteria.

Rank	Title	First author	Journal	Publication type	Publication year	Citations
1	Antibiotics for emerging pathogens	Fischbach MA	Science	Review	2009	1,227
2	Polyphenols as antimicrobial agents	Daglia M	Current Opinion in Biotechnology	Review	2012	779
3	Antimicrobial and phytochemical studies on 45 Indian medicinal plants against multi-drug resistant human pathogens	Ahmad I	Journal of Ethnopharmacology	Article	2001	556
4	Predictive compound accumulation rules yield a broad - spectrum antibiotic	Richter MF	Nature	Article	2017	402
5	Characterization of MexE-MexF-OprN, a positively regulated multidrug efflux system of *Pseudomonas aeruginosa*	Kohler T	Molecular Microbiology	Article	1997	384
6	Bacteria subsisting on antibiotics	Dantas G	Science	Article	2008	377
7	Anti-staphylococcal plant natural products	Gibbons S	Natural Product Reports	Review	2004	372
8	Antimicrobial activity of pomegranate (Punica granatum L.) fruit peels	Al-Zoreky NS	International Journal of Food Microbiology	Article	2009	361
9	Biosynthesis of silver nanoparticles from Tribulus terrestris and its antimicrobial activity: A novel biological approach	Gopinath V	Colloids and Surfaces B: Biointerfaces	Article	2012	315
10	Synthesis of silver nanoparticles using *Dioscorea* bulbifera tuber extract and evaluation of its synergistic potential in combination with antimicrobial agents	Ghosh S	International Journal of Nanomedicine	Article	2012	289

### Analysis of Co-cccurrence keywords

**FIGURE 8:**
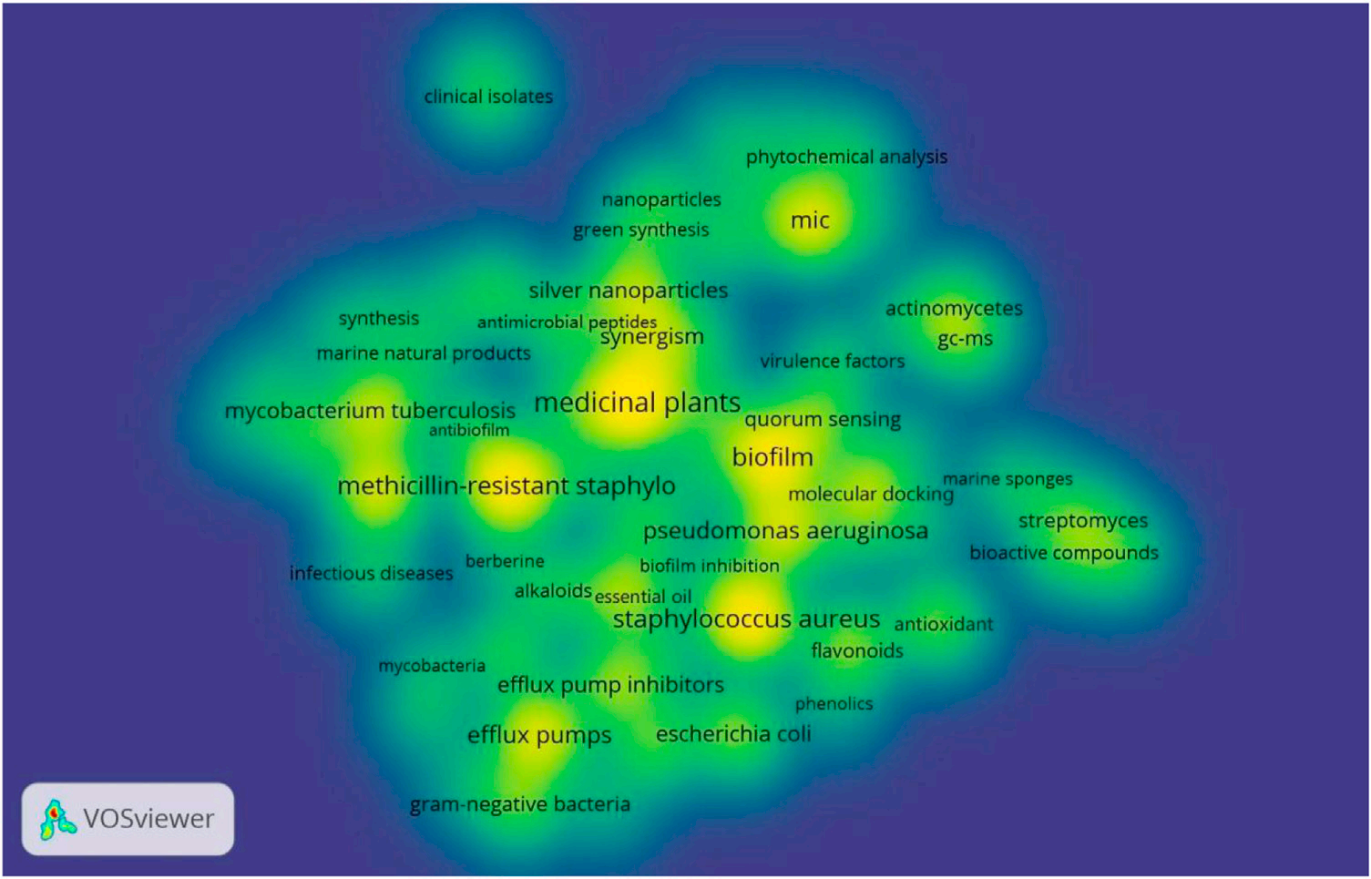
Density visualization map of keywords related to resistance of natural products to MDR bacteria.

Keywords are the concentration and refinement of the content of a paper, and keyword co-occurrence analysis is a commonly used scientometric method to track scientific development and identify research hotspots and emerging trends (Deng et al., 2020). There are 2,944 author keywords in 1,267 articles. The singular and plural forms, full and abbreviated forms, and synonyms of keywords were unified, and the keywords with general meaning were filtered out. Meanwhile, some keywords with high frequency but no analytical meaning were excluded, such as natural products, MDR, antibacterial activity and other search words. Only keywords with at least 6 co-occurrences were visualized, and 70 keywords reached this threshold in a density map (Figure 8). Among them, the top 20 keywords with frequency of occurrence are shown in Table 5. The most frequently occurring keywords were “medicinal plants”, “methicillin-resistant *Staphylococcus aureus*”, “*Staphylococcus aureus*”, “biofilm”, “minimum inhibitory concentration (MIC)”, “efflux pumps”, and so on.

**TABLE 5:** The 20 most frequently occurring keywords.

Rank	Keyword	Occurrence	TLS	Rank	Keyword	Occurrence	TLS
1	Medicinal plants	53	48	11	Molecular docking	24	24
2	Methicillin-resistant *Staphylococcus aureus*	48	42	12	Silver nanoparticles	23	15
3	*Staphylococcus aureus*	47	59	13	Efflux pump inhibitors	21	21
4	Biofilm	43	39	14	*Escherichia coli*	21	31
5	MIC	36	33	15	Flavonoids	21	26
6	Efflux pumps	34	35	16	Synergism	21	14
7	Tuberculosis	32	37	17	Antioxidant	19	13
8	*Pseudomonas aeruginosa*	31	43	18	Gram-negative bacteria	18	21
9	*Mycobacterium tuberculosis*	28	34	19	Antifungal	17	9
10	Essential oil	25	25	20	Quorum sensing	17	31

**FIGURE 9:**
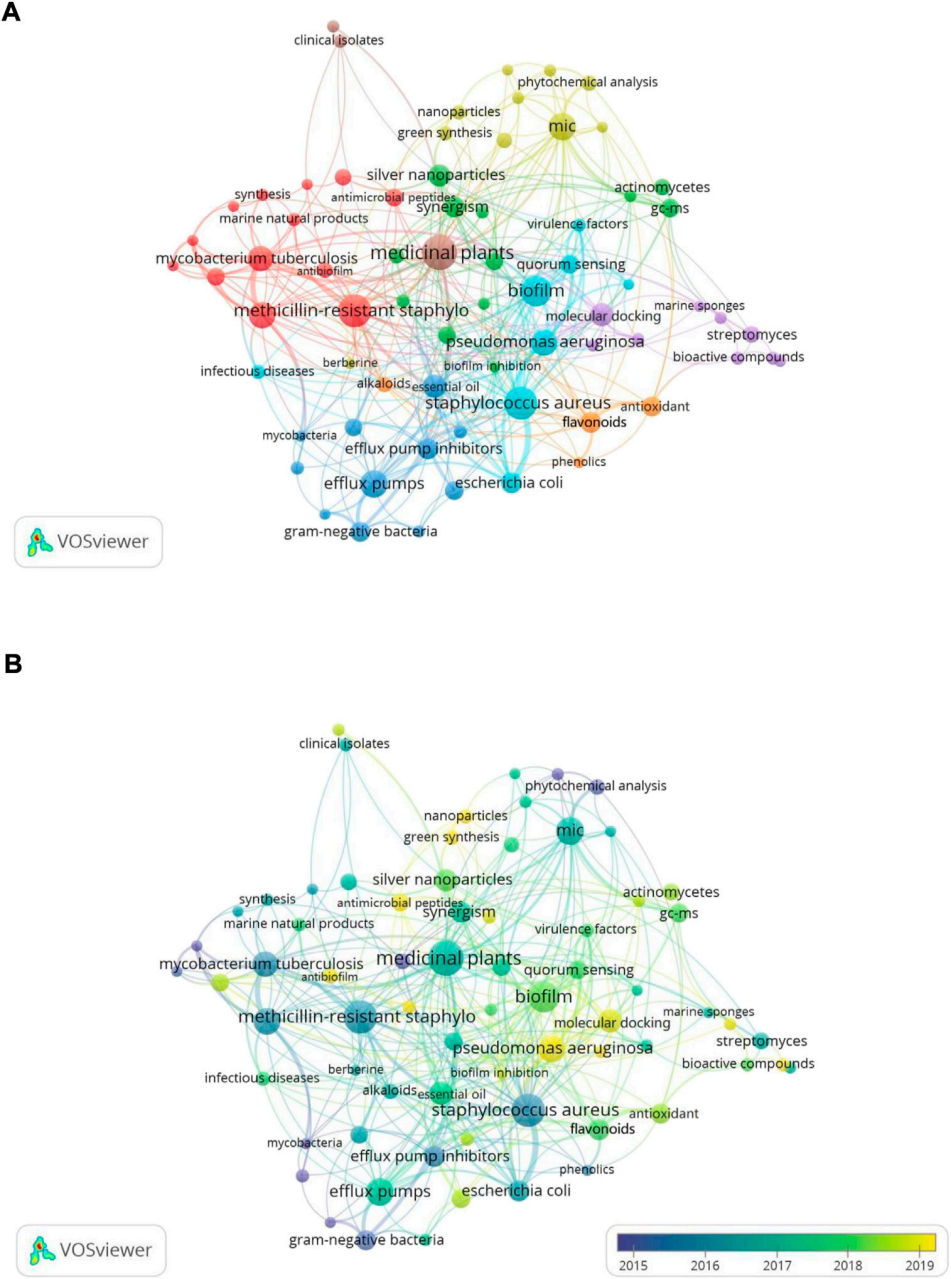
Network **(A)** and overlay **(B)** visualization map of keywords related to resistance of natural products to MDR bacteria.

As shown in Figure 9A, all these selected keywords were grouped into 8 clusters according to the co-occurrence relation network. After further sorting, these 8 clusters could be roughly divided into four main themes: “MDR bacteria” (red and light blue nodes), “mechanism of MDR” (dark blue nodes), “natural product sources and components” (brown and orange nodes), and “natural product development approach” (yellow, green, and purple nodes). In the theme of “MDR bacteria”, the frequently used keywords were “methicillin-resistant *Staphylococcus aureus*”, “*Staphylococcus aureus*”, “tuberculosis”, “*Pseudomonas aeruginosa*”, “*Mycobacterium tuberculosis*”, and “*Escherichia coli*”. In “mechanism for inhibiting MDR” theme, the prominent keywords were “efflux pumps” and “efflux pump inhibitors”. As for “natural product sources and components” theme, the primary keywords were “medicinal plants”, “flavonoids”, “antioxidant”, “alkaloids”, and “phenolics”. In the last theme, the major keywords were “MIC”, “molecular docking”, “silver nanoparticles”, and “synergism”.

In the overlay visualization map, the nodes for each keyword were colored based on their average publication year (Figure 9B). According to the color scale in the lower right corner, nodes identified in purple represented keywords that appeared before or around 2015, while keywords appearing around 2017 were identified in green, and keywords appearing around 2019 or after were coded in yellow. For example, “phytochemical analysis” and “gram-negative bacteria” appeared more frequently in the early stage. Keywords such as “*Pseudomonas aeruginosa*”, “nanoparticles”, “green synthesis”, “antimicrobial peptides”, “antibiofilm”, “antibiofilm activity”, “biosynthetic gene clusters”, and “molecular dynamics simulation” (the labels of the last three nodes are not shown in the map due to their location) were found in relatively recent years, implying that these topics have become popular and may become research hotspots of this field in coming years. Meanwhile, keywords in “natural product development approach” related clusters had the latest average publication year compared with other themes, indicating that research in this direction has received considerable attention recently.

## Discussion

According to the data of the WOS Core Collection database from 1997 to 2021, a total of 1,267 publications in 5 languages were retrieved, and the cumulative number of citations was 34,719. With the prevalence of bacterial resistance issues, studies on the antimicrobial activity of natural products against MDR bacteria has generated considerable exploratory interest, whether in terms of annual published documents or citation counts. Among the 95 countries/Regions involved in the publication of studies in this field, India was the most productive country. The United States ranked second in the total number of publications; however, it was the leading country in the total number of citations. Furthermore, the United States was the most active country in international cooperation and had close collaborations with India, China, Germany and Canada. In addition, China, India, Saudi Arabia, and Germany demonstrated active international cooperation as well. Therefore, combined with the quality and quantity of publications and international cooperation, the United States and India were dominant in this research field. In terms of institutional contribution, the University of Dschang had the largest output. As for the institution co-authorship analysis, the Regional University of Cariri, Federal University of Paraiba, Universidade Federal do Piaui, University of Dschang, and University of Porto showed active cooperation.

To some extent, the number of scientific papers published by an author in a certain field represents his contribution and activity in that field (Wu, et al., 2021b). Most researchers published only one or two papers, meaning that only a handful of authors made sustained contributions to the field. Three authors published more than 10 articles, namely Coutinho HDM, Kuete V and Gibbons S, with 33, 31 and 12 papers respectively. As for author co-authorship analysis, from the perspective of centrality, Coutinho HDM was located at a central position of the cooperating clusters. In author co-citation analysis, combined with the values of co-citations and TLS, Kuete V, Gibbons S and WHO were prominent in this field. Overall, the above results indicated that Coutinho HDM, Kuete V, and Gibbons S made significant contributions in this field. Based on the journal analysis, all papers included in this study were published in 505 journals. Among them, *Frontiers in Microbiology* (6.064, Q1), *Molecules* (4.927, Q2), *BMC Complementary and Alternative Medicine* (2.838, Q2), *Antibiotics-Basel* (5.222, Q1), and *Marine Drugs* (6.085, Q1) published the most paper in this research field. In addition to the number of publications, citation frequency is often considered as an important indicator to evaluate the influence of a journal (Wu, et al., 2021b). In the journal co-citation network graph, *Antimicrobial Agents and Chemotherapy* had the highest co-citations, followed by *Journal of Natural Products*, *Journal of Antimicrobial Chemotherapy*, *Journal of Bacteriology*, *Journal of Ethnopharmacology*, and *Proceedings of the National Academy of Sciences of the United States of America*. In general, there is a high probability that future achievements in the field will be published in the listed journals.

The top 10 highly cited papers were published between 1997 and 2017, with six published before 2010. In terms of research content, most of the top 10 most cited papers focused on the discovery of new antibiotics, especially natural product antibiotics of plants and natural products of plants were the primary source. The paper with the highest cited times, written by Fischbach and Walsh (2009) was published in *Science*, with 1,227 citations. It noted out that methods of new scaffold discovery, such as mining under-explored microbial niche for natural products, were promising approaches against MDR pathogens. The second most cited article was published in *Current Opinion in Biotechnology* by Daglia (2012). It discussed the synergistic effect of polyphenols in combination with conventional antibiotics against clinical MDR microorganisms. The research topics of five other papers also focused on the antibacterial activity of natural products of plant origin (Ahmad and Beg, 2001; Gibbons, 2004; Al-Zoreky, 2009; Ghosh, et al., 2012; Gopinath, et al., 2012), including two papers on synthesis of silver nanoparticles. The most recent article published in *Nature* with 402 citations. Richter et al. (2017) predicted compound accumulation rules in bacteria to aid in the discovery and development of novel antibacterial agents against Gram-negative bacteria. Co-cited references are documents in which two publications are cited together by other publications so that they can be considered as the background and foundation of research in a particular field (Ke, et al., 2020). Overall, the top 10 co-citations were focused on the following subjects: antibacterial activity of plant-derived natural products (Cowan, 1999; Kuete, 2010; Stermitz, et al., 2000); antibiotic discovery (efflux pump or cell wall inhibitors) (Ling, et al., 2015; Newman and Cragg, 2016; Stavri et al., 2007); antibiotic resistance (Davies and Davies, 2010); antibacterial discovery process, achievements, and challenges (Payne, et al., 2007); and MIC determination method (Eloff, 1998).

Keyword co-occurrence analysis is commonly used to highlight the frequently occurring keywords that can reveal the subject content and provide a reasonable description of the knowledge structure (Wu et al., 2021b). Meanwhile, the current hot topics and future directions can also be revealed (Guo et al., 2021). The analysis demonstrated that “medicinal plants”, “methicillin-resistant *Staphylococcus aureus*”, “*Staphylococcus aureus*”, “biofilm”, “MIC”, and “efflux pumps” were the most commonly used keywords in order, so these terms are presumed to be the hot spots in this field. Combining the formed clusters and all co-occurring keywords, among the related clusters of “MDR bacteria”, methicillin-resistant *Staphylococcus aureus*, *Pseudomonas aeruginosa* (Tasneem et al., 2022), *Mycobacterium tuberculosis* (Li et al., 2022), and *Escherichia coli* (Szmolka and Nagy, 2013) were the most frequently mentioned strains. In addition, *Acinetobacter baumannii* (Borges Duarte and Gonçalves Rodrigues, 2022) and *Klebsiella pneumoniae* (Ali et al., 2022) could be found in the co-occurring keywords. In “mechanism for inhibiting MDR” theme, suppression of the efflux pump was the primary mechanism studied (Nikaido, 2009). Moreover, anti-biofilm action was also a focus mechanism (Hathroubi, et al., 2017; Mishra, et al., 2020). As for “natural product sources and components” theme, medicinal plant-derived compounds gained significant attention as potential sources of new antimicrobial agents to combat MDR bacteria (Subramani et al., 2017). Besides, actinomycetes and marine natural products were also vital sources (Hou, et al., 2019; Jagannathan, et al., 2021; Zhu, et al., 2014). The notable active components were flavonoids (Song, et al., 2022), alkaloids and phenolics (Suganya, et al., 2022). Essential oil (Badescu, et al., 2022), polyphenols (Jubair, et al., 2021), and berberine (Yu, et al., 2005) were also included in the co-occurrence keywords. In “natural product development approach” theme, biosynthesis of nanoparticles, especially silver nanoparticles, had attracted much attention in order to develop new antibiotics (Anjum and Abbasi, 2016; Alavi, et al., 2022a; Alavi, et al., 2022b; Majoumouo, et al., 2019). Additionally, molecular docking was an impactful technology in drug design in this field (Miryala, et al., 2021). From the overlay visualization map, recent studies mainly focused on the theme of “natural product development approach”. Furthermore, the results showed that keywords such as “*Pseudomonas aeruginosa*”, “nanoparticles”, “green synthesis”, “antimicrobial peptides”, “antibiofilm”, “antibiofilm activity”, “biosynthetic gene clusters”, and “molecular dynamics simulation” had the latest average appearance year, indicating that these topics may become the research direction in the next few years.

In addition, the main limitations of our analysis are as follows: First, we only searched in the WOS database, and not in other databases such as PubMed, Embase, or Scopus. Therefore, the comprehensiveness of the data will be deficient to some extent. Second, we did not include relevant papers in 2022 because the data of this year are not yet complete enough to complete certain annual data comparison. As a result, some of the latest research progress may be missed. Third, all data in this study were extracted by software, unlike systematic reviews that are manually extracted by two or more independent authors. In consequence, the data used to derive the results in this paper may be biased.

## Conclusion

With the prevalence of bacterial resistance problems, interest in the antimicrobial activity of natural products against MDR bacteria has exploded during the past decades. In this study, the current research status and emerging global trends in this field were visualized using a scientometric method. A total of 1,267 related documents including 1,005 original articles and 262 reviews in a period from 1997 to 2021 were retrieved. India is the country with the most output, and the United States is the country with the highest total citations and the most active in international cooperation. The University of Dschang from Cameroon produced the most publications. Coutinho HDM, Kuete V, and Gibbons S had significant influence in this field, as they published a large number of articles and were co-cited in several more publications. *Frontiers in Microbiology* and *Antimicrobial Agents and Chemotherapy* were major journals with the highest number of publications and co-citations, respectively. Fischbach and Walsh published the most cited paper in Science with 1,227 citations. The content of most of the top 10 most cited papers focused on the discovery of new antibiotics, especially plant-derived natural product antibiotics. Based on keywords co-occurrence analysis, “medicinal plants”, “methicillin-resistant *Staphylococcus aureus*”, “*Staphylococcus aureus*”, “biofilm”, “MIC”, and “efflux pumps” were the most frequently used keywords, so these terms are presumed to be the current hot topics. All selected keywords could be roughly divided into four main themes: “MDR bacteria”, “mechanism for inhibiting MDR”, “natural product sources and components”, and “natural product development approach”, of which the last theme had attracted much attention in recent years. Methicillin-resistant *Staphylococcus aureus*, *Pseudomonas aeruginosa*, *Mycobacterium tuberculosis*, and *Escherichia coli* were the most commonly studied MDR strains, followed by *Acinetobacter baumannii* and *Klebsiella pneumoniae*. Inhibition of the efflux pump and anti-biofilm action were the primary mechanism for suppressing MDR. Medicinal plant-derived compounds such as flavonoids, alkaloids, and phenolics were important sources of novel antimicrobial agents against MDR bacteria, followed by actinomycetes and marine natural products. Besides, biosynthesis of nanoparticles, especially silver nanoparticles, was a new strategy to develop highly effective antibacterial agents. Molecular docking was also an important method in this field. Moreover, keywords such as “*Pseudomonas aeruginosa*”, “nanoparticles”, “green synthesis”, “antimicrobial peptides”, “antibiofilm”, “antibiofilm activity”, “biosynthetic gene clusters”, and “molecular dynamics simulation” had the latest average appearance year, indicating that these topics may become the research hot spots in the coming years. Overall, this study performed a scientometric analysis of the antibacterial activity of natural products against MDR bacteria from a holistic perspective. We hope it can provide an effective reference for the research points and future trends, not only for researchers already working in this field, but also for new researchers interested in this field.

## Data Availability

The original contributions presented in the study are included in the article/supplementary material, further inquiries can be directed to the corresponding author.
